# Implementation of an Internet-based psychological intervention for the treatment of mild depression in primary care: a hybrid effectiveness-implementation approach

**DOI:** 10.1186/s12889-026-27573-0

**Published:** 2026-06-03

**Authors:** Rosa Lorente-Català, Adoración Castro, Selene Fernández-Martínez, Esperanza Varela-Moreno, Gloria Guerrero-Pertiñez, Inés Forteza-Rey, Margalida Gili, Yolanda López-Del-Hoyo, Fermín Mayoral-Cleries, Javier García-Campayo, Azucena García-Palacios

**Affiliations:** 1 Department of Basic and Clinical Psychology, and Psychobiology, Universitat Jaume I, Castellon, Spain; 2 Health Research Institute of the Balearic Islands (IdiSBa), Hospital Universitario Son Espases, 07120 Palma, Spain; 3 Department of Psychology and Sociology, University of Zaragoza, Saragossa, Spain; 4 Mental Health Department, Biomedical Research Institute of Malaga (IBIMA), University Regional Hospital of Malaga, Malaga, Spain; 5 Research Institute of Health Sciences (IUNICS), University of Balearic Islands, 07122 Palma, Spain; 6 Institute for Health Research Aragón (IIS Aragón), 50009 Saragossa, Spain; 7 Network for Research On Chronicity, Primary Care, and Health Promotion (RICAPPS), Madrid, Spain; 8 CIBER Fisiopatología Obesidad y Nutrición (CIBEROBN), Instituto Carlos III, Madrid, Spain; 9 Unidad de Gestión Clínica de Salud Mental, Hospital Regional Universitario de Málaga, Malaga, Spain

**Keywords:** Depression, Internet-based interventions, Implementation research, Primary care

## Abstract

**Background:**

Depression is a significant global challenge. Particularly in Spain, depression in Primary Care (PC) settings is prevalent and inadequately addressed. Online interventions, such as "Smiling is Fun", an online Cognitive Behavioral program, offer promising solutions given its scalability and accessibility. However, successful implementation in real-world settings requires identifying and addressing contextual barriers. This study aims to implement "Smiling is Fun" while assessing its effectiveness and the determinants influencing its implementation.

**Methods:**

The study employed a Hybrid Effectiveness-Implementation Type II approach using a Stepped-Wedge Cluster Randomized-Controlled Trial methodology. Six sites were randomized into three clusters, each undergoing treatment conditions for 16 weeks. Patients were recruited by health professionals in PC settings.

**Results:**

The intervention effectively reduced depressive symptoms and improved positive affect. Participants generally had a positive attitude towards internet interventions, although there was a an increase in concerns about lack of direct professional contact.. Overall satisfaction among participants who completed the intervention was moderately high, but problems with treatment adherence were observed. Professionals demonstrated a willingness to adopt the program, with generally high readiness to implement the intervention. Nevertheless, some important barriers, such as high levels of stress, insufficient learning time, and personnel changes were highlighted.

**Conclusions:**

"Smiling is Fun" demostrated to be effective in addressing mental health states and depressive symptomatology, with positive perceptions from both professionals and patients. However, barriers need to be addressed, particularly in fostering inclusion in the PC workflow system and promoting patient adherence. Despite limitations, the study underscores the potential of online interventions to improve mental healthcare accessibility.

**Trial registration:**

https://clinicaltrials.gov/study/NCT05294614 (23/03/2022, retrospectively registered).

**Supplementary Information:**

The online version contains supplementary material available at 10.1186/s12889-026-27573-0.

## Introduction

Mental health has become a critical concern, ranking among the leading causes of Disability-Adjusted Life Years (DALYs) worldwide [1–3].

Depression stands out as one of the most significant conditions, currently the second leading cause of burden of disease globally and is expected to become the leading cause in 2030 [4, 5] Beyond its psychological impact, depression is associated with increased mortality and morbidity and substantial economic cost [6, 7], with even greater consequences when left untreated [8, 9].

Despite the availability of Evidence-Based Psychological Practices (EBPP), a substantial gap remains in effectively addressing depression at a global level [10]. This gap is largely attributed to challenges in translating evidence-based interventions into real-world settings, rather than a lack of effective treatments. In this context, Implementation Research (IR) provides a framework to examine the adoption of Evidence-Based Practices (EBP), focusing on the determinants that influence their uptake in routine care. [11].

Accordingly, this study adopts an implementation perspective, examining not only intervention effectiveness but also key factors influencing its uptake, including barriers and facilitators within primary care. The ultimate aim is to inform strategies to support the integration of digital mental health interventions into routine clinical practice [12, 13].

Specifically, in Spain, there is a high prevalence of depression, which has been further aggravated by the COVID-19 pandemic [14, 15]. The annual economic burden associated with depression in Spain is significant and has increased in recent years. The cost of this burden is estimated to be approximately 6.145 million euros per year, primarily attributed to its chronic nature and associated comorbidities [16]. Although this issue has been previously highlighted in Spain [17], satisfactory solutions have not yet been found, as only around 23% of individuals with depression receive minimally adequate treatment [18].

Primary Care (PC) settings serve as the frontline for detecting and managing most cases of depression [19]. In fact, depression is the most prevalent disorder seen in this setting in Spain [20]. Consequently, there is an urgent need to explore interventions aimed at addressing depression within health settings, particularly considering the unmet needs that have been identified [21].

One potential solution to the presented problem, promoting scalability and accessibility to intervention, is through online interventions [22]. Internet interventions are presented as a cost-effective and alternative solution to address the challenges associated with traditional psychotherapy [23]. In digital mental health, a key distinction exists between guided and unguided interventions. Meta-analyses consistently show that guided interventions achieve higher adherence, whereas self-guided formats, despite their higher scalability, face persistent challenges related to engagement and adherence [23, 24]. These challenges are especially critical in PC, where limited support and competing demands may further hinder uptake. Therefore, examining implementation processes, particularly engagement and adherence, is essential to ensure that self-guided interventions can be effectively integrated into routine care and respond to the growing demand for mental health services.

In this study, self-guided online interventions are proposed as a solution to address this issue in PC in Spain [25, 26], with the program “Smiling is fun” (in Spanish: “*Sonreír es divertido”*). "Smiling is Fun" is an online self-guided intervention designed to address low to moderate depression, backed by substantial evidence [27–29]. The results of these studies indicate that it is a promising candidate, showing positive effects not only on psychological symptoms but also at an economic level by promoting its scalability in an online format [30].

We have accumulated evidence from various Randomized Controlled Trials (RCT) of the Smiling is Fun program in different parts of Spain. Nevertheless, to meet the needs of the population, further steps are required to understand how this intervention functions in daily clinical settings and how it can become a standard treatment, going beyond randomized clinical trials with researchers’ support and resources [11]. In line with this objective, a specific study has been conducted in PC in Spain in three different Autonomous Communities (AC) to identify the factors influencing the use of the intervention and to promote its implementation to address the problem of depression.

While the determinants need further investigation and to be specified within this context, prior literature has provided valuable guidance for implementing strategies. A comprehensive set of 13 strategies has been identified for application in the ongoing study. These strategies were selected following Powell's compilation of 68 strategies [31], prioritizing Proctor's recommendations [32] and emphasizing Fixsen's core components [33]. The strategies were outlined in detail in the published protocol [34] including identifying and preparing champions, conducting educational meetings, etc. Throughout the study, careful consideration has been given to these strategies, with adaptations made as necessary to suit the specific context. In concordance, the translation and implementation of this program must be context-specific and consider the specific contextual barriers and facilitators. Some of the key factors that need to be addressed include the professionals involved, the beliefs about online interventions, the acceptance from patients, etc. [35, 36]. Furthermore, it is essential to identify determinant factors and barriers in order to successfully implement the program and maintain its effectiveness [37].

With the aim of addressing various research questions encompassing effectiveness and implementation outcomes, a Hybrid-effectiveness implementation study was conducted [38, 39], with the full protocol described previously[34]. Two primary outcomes were established in line with this design: (1) the effectiveness of Smiling is Fun in reducing depressive symptomatology in PC, and (2) implementation outcomes, focusing on identifying barriers and facilitators to adoption. Additionally, the study evaluated other implementation outcomes, including including acceptability, appropriateness, feasibility, fidelity, penetration, and sustainability.

## Methods

### Study design

A Hybrid Effectiveness-Implementation Type II approach was employed in PC facilities in Spain to address various research inquiries concerning both implementation and effectiveness issues in addressing depression [39].

This approach utilized a Stepped Wedge Cluster Randomized Controlled Trial (SW-CRT) methodology [40]. Three distinct sequences were established, randomized across six implementation sites grouped into three clusters. Subsequently, within each sequence, two PC facilities were randomly allocated. Each sequence consisted of eight clusters of the treatment condition (16 weeks) with a previous control condition of varying duration for each sequence. Control conditions comprised one cluster (two weeks), two clusters (four weeks), and three clusters (six weeks) for each variation of the sequence.

**Fig. 1:**
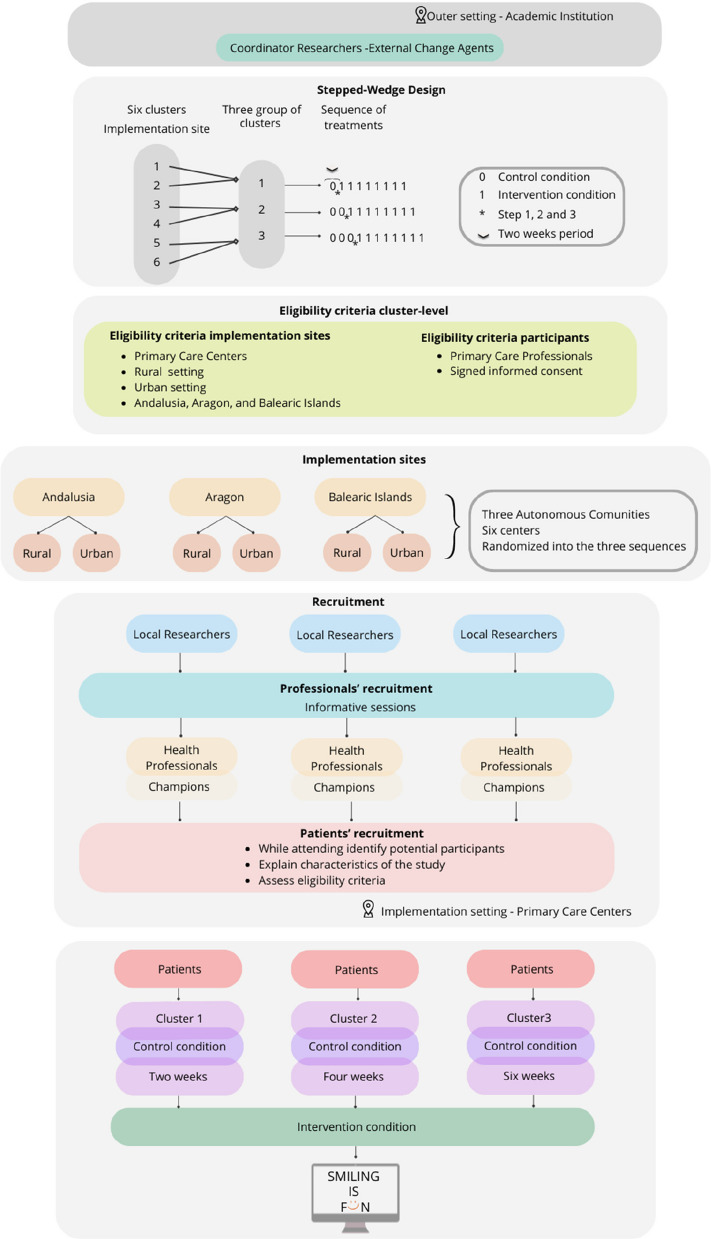
Design, eligibility criteria cluster-level, recruitment, and procedure

The detailed rationale and graphical representation of this design are comprehensively explained in the protocol study [34]. See the section of SW design in Fig. 1 for a brief description of the design and procedure.

The trial has been registered at ClinicalTrials.gov (NCT05294614). The study is reported following Standards for Reporting Implementation Studies Statement (StaRI) [41] regarding implementation and effectiveness outcomes. As this study employs a (SW-CRT) design, it also adheres to the CONSORT guidelines for reporting clinical trials, specifically the CONSORT extension for stepped-wedge cluster randomized trials [40].

### Participants, recruitment, and eligibility criteria

The study population is outlined in cluster and participant levels [40]. Information regarding all levels, settings, eligibility criteria, recruitment, and procedures is visually reflected in Fig. 1, with further detailed explanations available in the protocol [34].

At the cluster level, six implementation sites were predetermined, encompassing two PC settings (one rural and one urban) from each AC, including Andalusia, Aragon, and the Balearic Islands. Each PC was randomly assigned to one of the sequences established according to the SW-CRT. All health professionals attending PC were included as cluster-level participants. They provided informed consent. These professionals served as implementers in real-world settings and were responsible for patient recruitment.

**Fig. 2:**
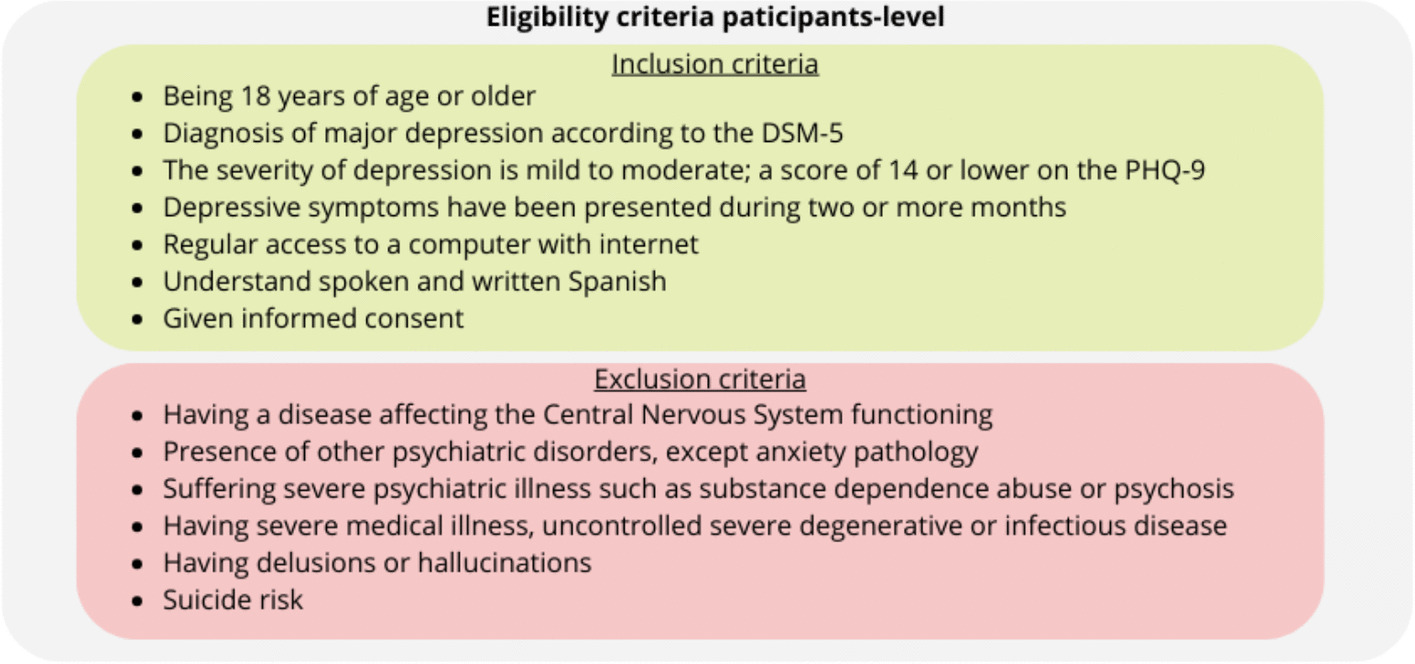
Eligibility criteria participants-level

At the participant level, inclusion and exclusion criteria for patients were established (refer to Fig. 2). Patients were recruited by health professionals, and after receiving study explanations and providing consent, they were assigned to one of the three conditions.

### Intervention

**Fig. 3:**
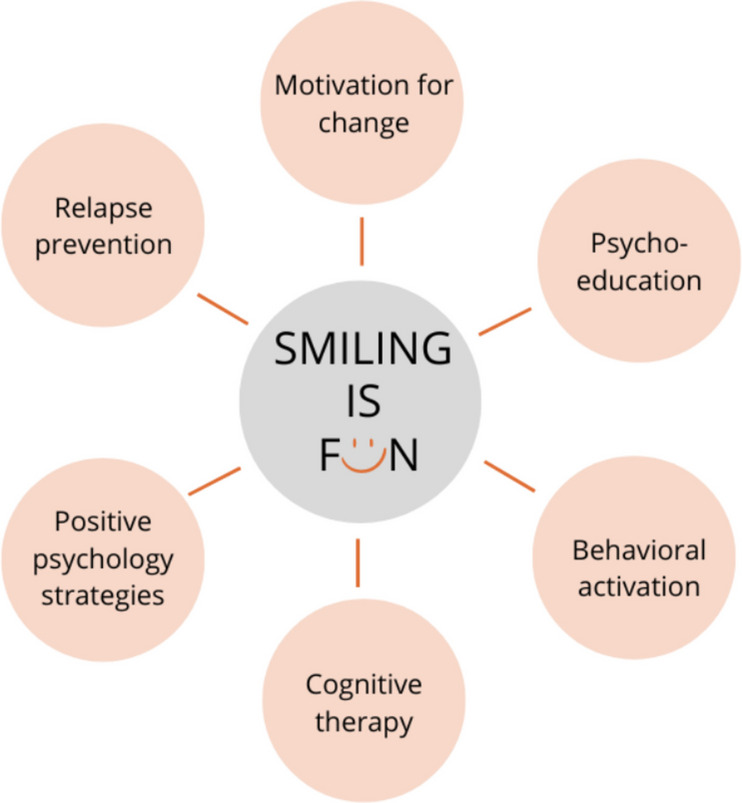
Smiling is Fun components

The applied innovation is "Smiling is Fun," which is a structured online self-guided intervention based on Cognitive Behavioral Therapy (CBT) designed to address depression disorders [29]. This intervention comprises six therapeutic components, including transdiagnostic components, cognitive behavioral therapy, and positive psychology (see Fig. 3. It consists of nine interactive modules and two introductory modules, presented sequentially. The modules are as follows: 1. Motivation for change; 2. Understanding emotional problems; 3. Sleep and medication management; 4. Learning to move; 5. Learning to be flexible; 6. Learning to enjoy; 7. Learning to live; 8. Living and learning; 9. From now on, what else…? The intervention is estimated to last eight to sixteen weeks. Upon completion of a module, the platform includes a review section to revisit the completed modules. For detailed information about the modules, including objectives, contents, and exercises, refer to Table A.1. of the protocol [34].

Each module provides psychoeducational information along with practical exercises, combining text with multimedia elements such as videos, audios, and images. Additionally, the intervention offers three tools: (1) a calendar for tracking the frequency of program use, completed and pending tasks; (2) a guided diary for participants to focus on the topics covered in the modules; and (3) a "How I am?" section graphically displaying the relationship between mood state and completed program tasks.

### Measures

The outcomes included in the study address effectiveness and implementation outcomes related to the implemented innovation. Detailed information is included in the protocol [34].

For the effectiveness outcomes, patients receiving the intervention were assessed on severity of depressive symtoms (Patient Health Questionnaire- 9; PHQ-9) [42, 43], the presence and valence of emotional affect (Positive and Negative Affect Schedule,PANAS-SF) [44, 45] and the severity on mobility, self-care, usual activities, pain/discomfort, anxiety and depression (EuroQol-5D-5L) [46].

Regarding the implementation outcomes, all level participants were considered including patients and implementers (professionals and managers).

The study employed a comprehensive set of implementation measures across several domains. Feasibility was assessed using the Feasibility of Intervention Measure (FIM) [47], which evaluates the extent to which the intervention can be successfully used. Acceptability was examined through multiple tools, including the Acceptability of Intervention Measure (AIM) [47], the Attitudes towards Psychological Online Interventions (APOI) [48], the Evidence-Based Practice Attitude Scale (EBPAS) [49, 50] and the Client Satisfaction Questionnaire, all aimed at capturing participants’ perceptions, attitudes, and satisfaction with the intervention. Appropriateness was measured with the Normalization MeAsure Development Questionnaire (NoMAD) [51, 52], which assesses the extent to which the intervention becomes integrated into routine practice, and the Intervention Appropriateness Measure (IAM) [47], which evaluates perceived suitability of the intervention. Fidelity was indexed by the number of completed modules, while penetration was reflected in the number of providers or managers who delivered the online intervention, indicating its integration within the service. Finally, sustainability was explored using the Barriers and Facilitators of Implementation (FBI) tool [34, 53], the Implementation Climate Scale (ICS) [54], and the Implementation Leadership Scale (ILS) [55], Organizational Readiness for Implementing Change (ORIC) [56], which together capture the contextual factors, strategic climate, leadership supporting the long-term implementation of evidence-based practices and he extent to which members of an organization are psychologically and behaviorally prepared to implement change.

Other measures included patients’ socio-demographic characteristics including gender, age, marital status, educational level, employment status, and income level. Demographics were also gathered from professionals: gender, age, qualifications, professional experience, time working in the department, time spent attending to patients, internet use and abilities, and expertise regarding online interventions.

### Sample size

The total sample size established in the protocol was 420 patients, with 140 designated for each AC. The calculation was performed based on SW designs, assuming a conservative effect size (0.25), a power of 0.80, and an alpha of 0.05. Following adjustment with a correction factor (DEsw) (this is a design effectt for SW-CRT, a correction factor used in sample size calculations) and accounting for an assumed experimental loss of 20%, it was determined that recruiting 70 participants per center would be required. Detailed explanations regarding the considerations made during the sample size calculation can be found in the study protocol [34].

### Data analysis

Analyses were conducted using the statistical package SPSS Statistics v.28. Descriptive analyses were performed to describe the sample characteristics, including sociodemographic variables, for the population assessed and included in the study.

In the protocol, ANOVA analyses were initially planned. However, normality has been tested using the Kolmogorov–Smirnov and it was found that the data were non-normally distributed for some variables. In such cases, it was established in the protocol to use non-parametric tests, specifically the Kruskal–Wallis test. Nonetheless, due to the significantly smaller sample size than expected, more robust methods were considered necessary. As a result, Intention-To-Treat (ITT) approach was conducted thorugh linear mixed models were employed with time as a factor, incorporating Bonferroni adjustments. Magnitude effect was reported according to Cohens’ d Effect Size.

Furthermore, non-parametric tests using Kruskal–Wallis test for all data except for PHQ-9, where the Friedman test was utilized, were conducted to confirm the findings.

Although module completion was recorded as an indicator of adherence (see Table 1), no formal analysis was conducted to examine the relationship between engagement with specific therapeutic components and effectiveness outcomes. This decision was primarily driven by the reduced sample size and the high rate of non-initiation, which limited the statistical power required for such analyses. Future research with larger samples should investigate whether completion of particular modules is differentially associated with improvements in depressive symptomatology or affect, which would allow for a more nuanced understanding of the active components driving therapeutic change.

**Table 1:** Characteristics of recruited patients

	**Total N**	**%**		**Total N**	**%**
**Marital stage**			**Employment status**		
Married or in a relationship	60	46.5%	Student	5	3,9%
Single	41	31.8%	Homemaker	4	3.1%
Separated or divorced	25	19.4%	Unemployed with benefits	17	13.12%
Widowed	3	2.3%	Unemployed without benefits	55	42.6%
N total	129	100%	Employed	37	28.7%
**Living**			Employed but on sick leave	7	5.4%
Own residence alone	14	10.9%	Retired	3	2.3%
Own residence with partner	15	11.6%	Permanently disabled	1	0.8%
Own residence with partner and/or children	53	41.1%	**N total**	129	100%
Family home	33	25.6%	**Income level**		
Other	14	10.9%	< MIS	9	7%
N Total	129	100%	1–2 MIS	42	32.6%
**Educational level**			2–4 MIS	8	32.6%
Not completed studies but knows how to read and write	3	2.3%	> 4 MIS	3	2.3%
Secondary school graduate	16	12.4%	N total	62	48.1%
Secondary studies	59	45.7%	**Reasons for abandonment**		
University studies	51	30.5%	Not need it	4	17.4%
N total	129	100%	Does not believe in its usefulness	1	4.3%
			Lack of time	6	26.1%
			Not comfortable with the program’s interface	2	8.7%
			Prefers personal interaction	2	8.7%
			Other	8	34.8%
			N total	23	100%
**Module Completion**					
0	45	39.1			
1	4	3.5			
2	8	7.0			
3	5	4.3			
4	12	10.4			
5	6	5.2			
6	3	2.6			
7	3	2.6			
8	3	2.6			
9	2	1.7			
10	24	20.9			
N total	115				

*N* sample size, *SD* Standard Deviation, *MIS* Minimum Interprofessional salary (< MIS = 900 euros; 1–2 MIS = 901 to 1800 euros; 2–4 MIS = 1801 to 3600; > 4MIS = more than 3601 euros)

## Results

### Patients

#### Demographic data

A total sample of 186 people were recruited, out of which 57 did not meet the criteria or declined to participate. Of these 57 participants; 27 did not meet the criteria established by PHQ-9, 11 did not answer after several attempts, 9 did not present diagnosis for depression, 7 of them finally were not interested in participating, and 3 lacked an appropriate internet device. Consequently, 129 participants were recruited and met the criteria. The mean age of the sample was 43.16 (SD = 11.79). Most of the sample were women (*N = *99, 76.7%) compared to a smaller number of men (*N = *30; 23.3%). The majority were married or in a relationship (*N = *60; 46.5%) and were living in their own house with their partner and/or children (*N = *53; 41.1%). The majority had completed secondary education (*N = *59; 45.7%) or university studies (*N = *51; 39.5%). Only 28.7% of the sample was actively working (*N = *7). Most of them were unemployed (*N = *72, 55.8%) and only 13.2% were receiving subsidies. The majority were in the first range of the minimum salary stipulated (*N = *42; 32.6%). Detailed characteristics of the patients recruited are reflected in Table 1.

#### Effectiveness outcomes

The effectiveness of the intervention was assessed by observing the depressive symptomatology with the PHQ-9 scale at three distinct points in time: initially when accessing the platform (PRE-1), after the control period (PRE-2), and upon intervention completion (POST). Specifically, a significant decrease of the depressive symptomatology was observed across all three time points (F_(2,112)_ = 44.47; *p* = < 0.001). Furthermore, there was a low to moderate effect size observed between PRE-1 and PRE-2 (d = 0.34), a moderate to large effect size between time PRE-2 and POST (d = 0.62), and a large effect size between PRE-1 and POST (d = 0.87).

Through PANAS, both positive affect and negative affect improvement were observed. Specifically, there was an increase in positive affect (F_(1,48)_ = 40.12; *p* < 0.001) and a decrease in negative affect (F_(1,52)_ = 29.51; *p* < 0.001).

Based on the EQ-5D, the general health status of the patients was assessed. This scale indicates three levels based on the severity of issues; no problem (level 1), some problem (level 2) and a lot of problems (level 3). Regarding the various subscales, mobility (F_(1,52)_ = 0.20; *p* = 0.88) and looking after myself (F_(1,56)_ = 2.26; *p* = 0.14) did not show significant variations, indicating no problems in these areas.

Significant improvements were observed in all other subscales from pre to post. Specifically, there was a significant decrease in reported problems with usual activities (F_(1,47)_ = 6.23; *p* = 0.01), having pain or discomfort (F_(1,33)_ = 6.82; *p* = 0.01), and feeling worried and sad or unhappy (F_(1,53)_ = 31.5; *p* < 0.001). Finally, a significant overall improvement was observed in the general health perceived by the patient on the day of the assessment (F_(1,52)_ = 12.28; *p* < 0.001).

Regarding the perception of the intervention (AIM, IAM and FIM), there was a no impact observed in the acceptability (AIM) of the intervention (F_(1,51)_ = 0.51; *p* = 0.47), the appropriateness (IAM) (F_(1,57)_ = 0.001; *p* = 0.97 and the feasibility (FIM) of the intervention (F_(1,35)_ = 2.48; *p* = 0.124).

The APOI was used to assess attitudes toward internet interventions. A non-significance decrease was observed in negative attitudes toward the efficacy and security of the intervention (Skepticism and Perception of Risk-SKE) (F_(1,64)_ = 0.04; *p* = 0.82), positive attitudes regarding the utility and credibility of the intervention (Confidence in Effectiveness-CON) (F_(1,72)_ = 0.21; *p* = 0.64), and positive attitudes related to the privacy gained through online interventions (Anonymity Benefits-ABE) (F_(1,51)_ = 1.09; *p* = 0.30). A significant increase was observed from pre to post in relation to concerns about the lack of professional contact (Technologization Threat-TET) (F_(1,51)_ = 7.45; *p* = 0.009). Overall, there was a non-significant increase in the total score of the APOI, reflecting the general acceptance of online interventions (F_(1,56)_ = 0.03; *p* = 0.86).

Considering the satisfaction of those who completed all the intervention, an average mean of 23.14 (Range = 8–32; SD = 8.76) was obtained in the CSQ-I questionnaire.

The results obtained are reflected in Table 2.

**Table 2:** Patient outcomes

	**Pre**		**Post**	**Pre-Post**				
				F	P	D		
PHQ9	14.71 (0.69)	12.20 (0.69)	6.06 (0.90)	44.47	** <.001**	0.34_PRE1-PRE2_	0.62_PRE2-POST_	0.87_PRE-POST_
PANAS-PA	17.69 (0.77)		26.31 (1.31)	40.12	** <.001**	0.91		
PANAS-NA	29.52 (0.95)		19.41 (1.69)	29.51	** <.001**	0.75		
AIM	4.20 (0.08)		4.09 (0.14)	0.515	0.47	0.10		
IAM	4.30 (0.08)		4.31 (0.14)	0.001	0.97	0.004		
FIM	4,21 (0.07)		4.41 (0.12)	2.48	0.12	0.26		
APOI SKE	2.32 (0.08)		2.28 (0.14)	0.04	0.82	0.02		
APOI CON	2.15 (0.09)		2.05 (0.17)	0.21	0.64	0.05		
APOI TET	3.20 (0.07)		3.57 (0.12)	7.45	**0.009**	0.38		
APOI ABE	3.06 (0.10)		2.86 (0.17)	1.09	0.30	0.14		
APOI total	42.94 (0.83)		43.20 (1.44)	0.03	0.86	0.02		
EUROQOL mobility	1.25 (0.05)		1.26 (0.09)	0.02	0.88	0.01		
EUROQOL looking after myself	1.29 (0.05)		1.13 (0.09)	2.25	0.13	0.19		
EUROQOL doing usual activities	1.83 (0.06)		1.54 (0.11)	6.23	**0.01**	0.36		
EUROQOL having pain or discomfort	1.84 (0.07)		1.51 (0.12)	6.82	**0.01**	0.45		
EUROQOL feeling worried sad or unhappy	2.31 (0.06)		1.62 (0.11)	31.54	** <.001**	0.77		
EUROQOL today	45.77 (2.19)		59.95 (3.81)	12.27	** <.001**	0.48		

*ABE* Anonymity Benefits, *AIM* Acceptability of Intervention Measure, *APOI* Attitudes towards Psychological Online Interventions, *CON* Confidence in Effectiveness, *FIM* Feasibility of Intervention Measure, *IAM* Intervention Appropriateness Measure, *PANAS-NA* Positive and Negative Affect Schedule-Negative Affect, *PANAS-PA* Positive and Negative Affect Schedule-Positive Affect, *PHQ-9* Patient Health Questionnaire- 9, *SKE* Skepticism and Perception of Risk, *TET* Technologization Threat

Bold values indicate statistically significant results (*p* < 0.05)

### Professionals

#### Demographic data

Of the 86 recruited professionals (Balearic Island = 28; Andalusia = 34; Arago*N = *24), only 57 (Balearic Island = 25; Andalusia = 18; Arago*N = *14) were successfully contacted for a telephone interview and provided access to online intervention. Among them, 45 were women (78.9%) and 12 were men (21.1%) with an average age of 45.58 (SD = 11.29; range 25–65). Of the recruited professionals, 36 were medical doctors (63.2%) and 11 nurses (19.3%), while information regarding the professional background of 10 individuals was not available. The absence of psychologists in the professional sample reflects the structure of the Spanish PC system. In Spain, PC is staffed primarily by general practitioners and nurses; clinical psychologists are generally not part of the standard PC team in the public health system, being instead located in secondary care or specialized mental health units. The characteristics of the professionals are reflected in Table 3.

**Table 3:** Professionals’ information

	**N**	**%(N)**		**N**	**% (N)**
**Qualification**			**New patients**		
Bachelor’s degree	1	1.8	< 20	28	49.1
Degree	12	21.1	20–30	15	26.3
Master	6	10.5	30–40	7	12.3
PhD	3	5.3	> 40	5	8.8
Specialization	30	52.6	N total	55	96.5
N total	52	91.2	**Internet use**		
**Profession**			Mostly every day	51	89.5
Doctor	36	63.2	Several times per week	5	8.8
Nurse	11	19.3	N total	56	98.2
N total	47	82.5	**Internet abilities**		
**Experience**			Really good	7	12.3
1–2 years	1	1.8	Good	22	38.6
3–5 years	8	14	Half	22	38.6
6–10 years	6	10.5	Poor	6	10.5
11–15 years	4	7	N total	57	
> 15 years	37	64.9	**Seen online interventions**		
N total	56	98.2	Yes	9	15.8
**Time department**			No	47	82.5
< 1 year	13	22.8	Do not know	1	1.8
1–2 years	11	19.3	N total	57	
3–5 years	15	26.3	**Trained to apply online intervention**		
6–10 years	7	12.3	Yes	4	7
11–15 years	3	5.3	No	53	93
> 15 years	8	14	N total	57	
N total	57	100	**Used online intervention**		
			Yes	4	7
			No	53	93
			N total	57	

Most of the professionals had extensive experience, with over fifteen years in the profession (*N = *37; 64.9%). However, their time in the department was shorter, with most having been in their current department for 3 to 5 years (*N = *15; 26.3%), followed by 13 with less than 1 year and 11 with between 1 and 2 years.

#### Implementation outcomes

Regarding the affluence of patients, the majority claimed to see fewer than 20 new patients per month (*N = *28; 49.1%). In terms of attendance, professionals estimated a mean of 13.16 min per patients (SD = 10.53; range 6–60).

Regarding internet usage, the majority reported using it every day (*N = *51;89.5%) and expressed either good or average abilities in using the internet (in both cases *N = *22;38.6%). The internet abilities are in congruence with the technological profile assessment, where they got a mean of 2.15 (SD = 0.54), being 2 categorized as normal. However, regarding internet interventions, most of them had not seen (*N = *47; 82.5%), received training, or used an online intervention (in both cases *N = *53; 93%).

Professionals’ willingness to adopt innovations on EBP (EBPAS) was demonstrated with a mean score of 3.77 out of 5 (Range = 2.38–4.38; SD = 0.39; *N = *38).

Regarding the climate for implementing (ICS) the intervention Smiling is Fun, the highest score was observed in the team’s focus on EBP with a mean of 3.09 (Range = 1–4; SD = 0.74). Other factors included providing educational support for implementation (Mea*N = *2.71; Range = 0.33–4; SD = 0.92), recognition of mental health workers for implementing the program (Mea*N = *2.47; Range = 0.67–3.67; SD = 0.81) and selecting mental health professionals for their openness (Mea*N = *2.33; Range = 0–4; SD = 1.2). The lowest ranked factors were selecting based solely on prior experience in the program or similar ones (Mea*N = *1.63; Range = 0–4; SD = 1.3) and offering rewards to professionals for program implementation (Mea*N = *0.91; Range = 0–3; SD = 1.07). The overall mean score for the scale was 2.19 (Range = 0.44–3.44; SD = 0.78). Additionally, the readiness of the professionals within PC settings to implement the intervention was assessed using the ORIC scale, yielding an average of 4.07 (range = 1.5–5; SD = 0.87).

Leadership style was also assessed using the ILS scale, with the highest mean observed in the Supportive attitudes at 3.02 (Range = 1–4; SD = 0.74). This was followed sequentially by Knowledgeable (Mea*N = *2.80; Range = 0.67–4; SD = 0.80), Perseverant (Mea*N = *2.76; Range = 0.75–4; SD = 0.84), and Proactive (Mea*N = *2.45; Range = 0–4; SD = 1.94). The average total score for the scale was 2.76 (Range = 0.75–4; SD = 0.76).

Based on the list of barriers and facilitators (Appendix 2; Table 4; List of barriers and facilitators) listed in the FBI questionnaire, professionals reported prior to implementation which aspects they believed could hinder or facilitate the progress of implementing the intervention “Smiling is Fun,” even if only to a small extent. Once the intervention was implemented, professionals were asked again which aspects finally interfered with or facilitated the process. The option “other” was offered in case professionals were uncertain or preferred not to answer.

Barriers more frequently pointed out before implementation included frequent changes in staff (*N = *32; 86.5%), insufficient time provided for learning and implementing the new program (*N = *29; 78.4%), and high levels of stress (*N = *28; 75.7%). After implementation, high levels of stress (*N = *19; 70.4%), lack of integration of the program into medical records (*N = *19; 70.4%), and a gap between professionals who attended the training and those who did not (*N = *18; 66.7%) were identified as prominent barriers.

As for facilitators highlighted before implementation, they included leader support for the intervention (*N = *34; 91.9%), organizational capacity (*N = *33; 89.2%), and reporting on patient outcomes and progress (*N = *33; 89.2%). After implementation, the most valued facilitators were the intervention’s alignment with the target population (*N = *22; 81.5%), perceived effectiveness of the intervention (*N = *21; 77.8%) and reporting on patient outcomes and progress (*N = *21; 77.8%).

Based on the set of scales AIM, IAM and FIM, a no impact has been observed in the acceptability (F_(1,43)_ = 2.44; *p* = 0.126; N_PRE_ = 4.45, SE_PRE_ = 0.01; N_POST_ = 4.22, SE_POST_ = 0.11), appropriateness (F_(1,34)_ = 7.8; *p* = 0.08; N_PRE_ = 4.56, SE_PRE_ = 0.11; N_POST_ = 4.20, SE_POST_ = 0.11) and feasibility (F_(1,44)_ = 3,49; *p* = 0.68; N_PRE_ = 4.22, SE_PRE_ = 0.11; N_POST_ = 3.91, SE_POST_ = 0.12) of the intervention perceived by professionals.

## Discussion

Depression needs to be addressed, and PC, which is established as the first step in public healthcare to receive assistance, offers the opportunity to tackle this issue in Spain [57]. With the primary objective of enhancing the population’s accessibility to EBPP, a Hybrid Effectiveness-Implementation Type II using SW-RCT approach was conducted. The study aimed to implement the program ‘Smiling is Fun’ to address mild to moderate depression.

One of the goals of the study was to evaluate the effectiveness of Smiling is Fun in addressing depression among participants. The findings indicate a significant reduction in depressive symptomatology with a large effect size from pre to post. The observed decrease in depressive symptoms aligns with existing literature highlighting the effectiveness of Smiling is Fun [29, 58] and other CBT online interventions in reducing depression [23, 24].

In addition to reductions in depressive symptoms, our findings also revealed significant improvements in positive affect and reductions in negative affect. This dual effect on affective states is consistent with the broader goal of promoting overall well-being and emotional resilience. By targeting both depressive symptoms and affective states, online interventions have the potential to offer comprehensive support for individuals experiencing mental health challenges [59]. The effects of Smiling is Fun are consistent with previous studies that focus on the effects of positive psychology components that build the intervention [27, 28].

Furthermore, a general improvement in the health status of the participants was observed. Specifically, the EQ-5D revealed a decrease in problematic areas related to depression symptomatology, including not doing the daily activities, having pain or discomfort, and having emotional problems related to worry, sadness or unhappiness.

Consistent with previous research [10, 60], our findings suggest that online platforms can effectively deliver EBPP, underscoring the potential of online interventions as a viable approach for addressing depression and general mental health, particularly considering the convenience and accessibility they offer to individuals seeking support [61]. Once the effectiveness of the intervention was established, a parallel objective was to assess implementation outcomes influencing this process. A particularly relevant finding is that 45 out of 129 recruited participants (34.9%) did not initiate any intervention module. This high rate of non-engagement prior to treatment onset represents a critical implementation challenge distinct from dropout during treatment and likely reflects barriers at the transition between referral and active use, such as limited motivation or difficulties accessing the platform. This pattern is consistent with previous research indicating that non-initiation is a persistent challenge in self-guided digital interventions, with engagement barriers emerging even before treatment begins [62]. Importantly, meta-analytic evidence and empirical trials also suggest that brief supportive contacts as follow-up calls can improve adherence and completion rates in self-guided treatments. These findings underscore the need to explore implementation strategies that incorporate low-intensity support mechanisms to enhance adherence, which could be especially valuable in primary care settings where intensive human guidance may not be feasible [63].

Regarding the acceptability, appropriateness, and feasibility, both patients and professionals perceived it as moderately to highly favorable. These perceptions remained relatively stable throughout the implementation process. The lack of significant changes in acceptability, appropriateness, and feasibility suggests that participants' overall perceptions of the intervention remained steady. Despite not experiencing a significant increase, and rather observing a non-significant decrease in most cases, this consistency implies that the intervention maintained its perceived value and relevance.

Examining attitudes in patients toward internet interventions using the APOI revealed a broader perspective. Although skepticism and perceived risk and confidence in effectiveness were moderate, participants also demonstrated moderate levels on finding anonymity beneficial. While there were no significant changes, there were indications of pre-established attitudes that could hinder implementation. Addressing these hesitations is crucial for enhancing acceptance and uptake of internet interventions.

A notable concern was the lack of professional contact, evident from the significant increase in the Technologization Threat. This emphasizes the need to balance accessibility and the provision of direct interaction with healthcare professionals. This observation aligns with the existing literature, which suggests that interventions with minimal professional enrolment may lead to higher rates of adherence and effectiveness by addressing these beliefs [64]. Despite these perceptions, participants who completed the intervention reported high levels of satisfaction.

Fidelity of the implementation was determined by the number of modules completed. It is important to note that there is a need for further development to address this concern, as not all participants completed the entire intervention. Once more, the literature underscores lower rates of adherence in self-applied interventions, often attributed to participants' perceived lack of time to engage fully in the intervention themselves [65]. This constraint can significantly impact module completion rates.

Regarding sustainability, the climate for implementation using the ICS scale revealed several key factors influencing professionals' readiness to implement the intervention. The emphasis on the team's focus on EBPP suggests a supportive organizational culture for innovation and change. However, areas for improvement include providing educational support for implementation and recognizing mental health workers for their efforts, which can enhance motivation and engagement in the implementation process.

The readiness of professionals within PC settings to implement the intervention, as measured by the ORIC scale, indicates a high level of readiness overall. This suggests that professionals perceive themselves as capable and prepared to integrate the intervention into their practice, which is essential for successful implementation and sustainability.

The leadership style exhibited by professionals, as measured by the ILS scale, reflects a predominantly supportive approach, followed by knowledgeable, perseverant, and proactive attitudes. A supportive leadership style is essential to foster a positive organizational climate and facilitate the implementation process by providing guidance, encouragement, and resources to support staff members. The relatively high scores across all leadership dimensions indicate a favorable leadership context for implementing the intervention.

So it seems that PC settings could offer an adequate context to implement the intervention [66]. Nevertheless, more precise information is necessary, such as identifying barriers and facilitators for implementation which is essential to understand the contextual factors that can hinder or support the successful adoption of new interventions. Before implementation, professionals identified frequent changes in staff, insufficient time for learning and implementation, and high levels of stress as prominent barriers. Other barriers included lack of personnel and difficulties in following the process of implementation as established. These factors highlight the challenges inherent in introducing new interventions within dynamic and demanding healthcare environments.

After implementation, high levels of stress, lack of integration of the program into medical records, and disparities between trained and untrained professionals emerged as persistent barriers. These findings underscore the importance of ongoing support and resources to address stressors and promote the integration of interventions into existing workflows.

On the other hand, leader support for the intervention, organizational capacity, and reporting on patient outcomes and progress were identified as significant facilitators before implementation. These factors emphasize the critical role of leadership in championing the intervention, fostering a supportive organizational culture, and promoting accountability and transparency in implementation efforts.

It is important to note that there was a substantial drop from the 86 professionals initially recruited to 57 who were included in the analysis, with additional missing data across the different measures. This attrition appears to be directly related to some of the barriers identified before and after implementation, such as high levels of stress, insufficient time for learning and implementation, frequent staff changes, and lack of integration of the program into existing workflows. These challenges likely affected professional engagement, highlighting how contextual and organizational factors can impact participation and, ultimately, the sustainability of digital interventions in primary care. Addressing these barriers will be critical for maintaining involvement and promoting successful long-term adoption.

The implementation barriers are therefore not merely contextual factors but active determinants of clinical outcomes. Addressing them is essential not only to improve the sustainability of the intervention but to fully realize its therapeutic potential, as the effectiveness of even well-evidenced interventions is ultimately constrained by the conditions under which they are delivered. This integrated perspective is central to the value of hybrid effectiveness-implementation designs [38]

Following implementation, the intervention's alignment with the target population, perceived effectiveness, and continued reporting on patient outcomes emerged as key facilitators. These findings highlight the importance of maintaining alignment with organizational goals and priorities, demonstrating the intervention's effectiveness, and monitoring progress to sustain engagement and momentum over time.

Overall, the findings highlight the multifaceted nature of implementation processes within PC settings, where leadership style, organizational factors, and contextual barriers and facilitators intersect to shape the success of intervention efforts. By addressing identified barriers and leveraging facilitators, healthcare organizations can optimize their capacity to effectively implement and sustain interventions like Smiling is Fun, ultimately enhancing the delivery of mental healthcare within primary care settings.

In order to address some of the detected main barriers, new strategies need to be developed and tested such as the incorporation of online educational resources for professionals or addressing stress levels of health professionals [67].

Prior to implementation, a set of 13 strategies were designed and described [34]. However, during the implementation process, some difficulties arose, resulting in the partial or non-execution of certain strategies. For instance, financial strategies involving economic reinforcement per patient for professionals to promote adoption and penetration were hindered by the funder.

Another pre-established strategy that faced challenges was reminding clinicians about the recruitment and the study itself. Promotion of the implementation was hindered because physical visits to PC settings by local researchers made it difficult to contact professionals. However, midway through the implementation process, the identified health professional champions were called and interviewed. One significant barrier they identified was the overload of studies being conducted in the different centers, causing them to forget this particular one. In fact, when contacting professionals to conduct post-questionnaires, a notable percentage did not remember the project. Strategies in this regard need refinement and adaptation to the specific situation.

Additionally, another partially implemented strategy involved using advisory and work groups to qualitatively assess professionals. As mentioned earlier, champions of each PC center were assessed once. However, further assessment is needed, including a broader profile of professionals.

### Limitations

While this study presents several advantages, it should be interpreted within the context of its limitations. First, the small sample size and the recruitment problems restrict the generalizability of its findings. ITT approach were used to overcome these difficulties, nevertheless testing hypothesis should be interpreted cautiously. Regarding sample size should be noted that it was not possible to determine the proportion of eligible patients that were not invited to participate. Adherence issues were observed throughout the study, which may have impacted the consistency and reliability of the results. Furthermore, it was not possible to formally examine the relationship between engagement with specific intervention modules and clinical outcomes, which limits the conclusions that can be drawn about the therapeutic mechanisms underlying the observed improvements Additionally, the absence of recorded technical support hinders the assessment of the resources required for implementation and is especially related to economical and personal costs. Although an ITT approach was applied, satisfaction outcomes were only available for participants who completed the intervention. It is important to note that participants with lower levels of engagement were less likely to complete post-assessment measure. Therefore, satisfaction results may be biased toward participants with higher engagement.

An important limitation of the present study is that no formal analysis was conducted to examine the relationship between professional-level factors and patient outcomes. Nevertheless, it is plausible that barriers identified by professionals — particularly high stress levels, insufficient training, and lack of program integration into existing workflows — may have contributed to the low adherence and non-initiation rates observed among patients, as inner setting factors are recognized as key determinants of patient engagement in IR frameworks [68]. Future studies should formally examine these multilevel relationships, for instance through multilevel modelling linking professional barriers to patient adherence and clinical outcomes.

The study encountered significant challenges during its execution due to the unforeseen onset of the COVID-19 pandemic. This global health crisis resulted in substantial disruptions, particularly in the overload experienced by professionals and service attendance. Therefore, any adaptations or repetitions of this study should account for these unique circumstances.

Moreover, the absence of follow-up due to insufficient data hampers our ability to assess the sustainability of the observed changes over time.

## Conclusion

In conclusion, while the study demonstrates promising outcomes regarding the effectiveness of the 'Smiling is Fun' intervention in addressing depression and enhancing overall well-being, it is essential to acknowledge the complexity of implementing such interventions within PC settings. The identified barriers and facilitators underscore the need for ongoing refinement and adaptation of strategies to optimize implementation success. Despite its limitations, this study contributes valuable insights into the potential of online interventions to augment mental health care accessibility and delivery. Moving forward, continued research and development efforts are warranted to address challenges and ensure the sustained impact of interventions like 'Smiling is Fun' in real-world healthcare contexts.

## Supplementary Information

Supplementary Material 1: Appendix 1. StaRI Checklist

Supplementary Material 2: Appendix 2. Table 4. List of barriers and facilitators.

Supplementary Material 3.

## Data Availability

The datasets used and/or analyzed during the current study are available from the corresponding author.
