# Supplementary material for: Implementation of an Internet-based psychological intervention for the treatment of mild depression in primary care: a hybrid effectiveness-implementation approach
Source: BMC Public Health. 2026 Jun 3;26:2301. doi: 10.1186/s12889-026-27573-0 (PMC13449318; doi:10.1186/s12889-026-27573-0)
Supplement: Supplementary file 2 [file 12889_2026_27573_MOESM2_ESM.pdf]

Appendix 1.

Table 4. Barriers and facilitators

	PRE						POST					
**Barrier**	Yes		No		Other		Yes		No		Other	
	N	%	N	%	N	%	N	%	N	%	N	%
Change of administrator	23	62.2	10	27	4	8.5	2	7.4	16	55.6	10	37
Frequent change of staff	32	86.5	3	8.1	2	5.4	15	55.6	8	29.6	4	14.8
Lack of conviction about the effectiveness of the program	25	67.6	11	29.7	1	2.7	12	44.4	11	40.7	4	14.8
Insufficient time provided for learning and implementing the new program	29	78.4	4	10.8	4	10.8	16	59.3	8	29.6	3	11.1
Lack of leadership	22	59.5	12	32.4	3	8.1	10	37	12	44.4	5	18.5
Unprepared staff	24	64.9	11	29.7	2	5.4	11	40.7	12	44.4	4	14.8
High levels of stress	28	75.7	6	16.2	3	6.4	19	70.4	4	14.8	4	14.8
Insufficient number of patients who can benefit from the intervention	17	45.9	18	48.6	2	5.4	5	18.5	18	66.7	4	14.8

Too many patients to assign to the intervention	13	35.1	22	59.5	2	4.3	5	18.5	19	70.4	3	11.1
Gap between professionals who attended the training and those who did not	19	40.4	12	32.4	6	16.2	18	66.7	3	11.1	6	22.2
Patients accustomed to the treatment they have already received and resistant to change	26	70.3	9	19.1	2	5.4	9	33.3	15	55.6	3	11.1
Difficulties in following the process and procedures for implementing the intervention program	24	64.9	10	27	3	8.1	15	55.6	10	37	2	7.4
Resistance to change from professionals	21	56.8	12	32.4	4	10.8	14	51.9	11	40.7	2	7.4
Conflict between the intervention and the organization's mission/culture	14	37.8	17	45.9	6	12.8	3	11.1	19	70.4	5	18.5
Lack of prioritization of the intervention	20	54.1	14	37.8	3	6.4	13	48.1	10	37	4	14.8
Lack of administrative infrastructure	23	62.2	13	35.1	1	2.7	12	44.4	12	44.4	3	11.1
Lack of financial resources	21	56.8	13	35.1	3	8.1	7	25.9	13	48.1	7	25.9
Lack of personnel	26	70.3	8	21.6	3	8.1	14	51.9	8	29.6	5	18.5
Lack of technical resources	22	59.5	13	35.1	2	5.4	10	37	12	44.4	5	10.6
Resistance and non-participation of patients	22	46.8	13	35.1	2	5.4	11	40.7	13	48.1	3	11.1

Low attendance of patients at appointments	11	29.7	26	70.3	-	-	5	18.5	17	63	5	10.6
The organization's mission is not clear	8	21.6	25	67.6	4	10.8	4	14.8	18	66.7	5	18.5
Low cohesion within the organization	12	32.4	23	62.2	2	5.4	6	22.2	15	55.6	6	22.2
The intervention does not fit the type of population being treated	8	21.6	27	73	2	5.4	2	7.4	23	85.2	2	4.3
Lack of informatic personnel	16	43.2	18	48.6	3	8.1	8	29.6	12	44.4	7	25.9
Lack of integration of the program into the medical records	19	51.4	13	35.1	5	13.5	19	70.4	6	22.2	2	7.4
Issues with internet access	18	48.6	17	45.9	2	5.4	9	33.3	16	59.3	2	7.4
Problems with the software	20	54.1	15	40.5	2	5.4	4	14.8	18	66.7	5	18.5
	**PRE**						**POST**					
**Facilitator**	Yes		No		Other		Yes		No		Other	
	N	%	N	%	N	%	N	%	N	%	N	%
The intervention aligns well with the organization's mission	33	70.2	-	-	4	8.5	20	74.1	2	7.4	5	18.5

The organization supports/prioritizes the intervention	32	86.5	1	2.7	4	10.8	17	63	5	18.5	5	18.5
Leaders support the intervention	34	91.9	-	-	3	8.1	20	74.1	7	25.9	7	25.9
Organizational capacity	33	89.2	2	5.4	2	5.4	13	48.1	9	33.3	5	18.5
Organizational stability	29	78.4	5	13.5	3	8.1	11	40.7	9	33.3	7	25.9
Professional experience	32	86.5	3	8.1	2	5.4	19	70.4	5	18.5	3	11.1
Training received on the intervention	29	78.4	5	13.5	3	8.1	17	63	5	18.5	5	18.5
Administrative capacity and infrastructure	30	81.1	5	13.5	2	5.4	11	40.7	8	29.6	8	29.6
Perceived effectiveness of the intervention	33	89.2	2	5.4	2	5.4	21	77.8	2	7.4	4	14.8
Ongoing evaluation of the intervention	32	86.5	3	8.1	2	5.4	16	59.3	7	25.9	4	8.5
Novelty of the intervention	31	83.8	2	5.4	4	10.8	18	66.7	6	22.2	3	11.1
The intervention fits with the target population	32	86.5	1	2.7	4	10.8	22	81.5	1	3.7	4	14.8
Possibility of collaborating with another organization to implement the intervention	30	81.1	4	10.8	3	8.1	12	44.4	7	25.9	8	29.6

Providing incentives/reinforcements to patients for using the program	28	75.7	6	16.2	3	8.1	11	40.7	8	29.6	8	29.6
Continuous support for implementing the intervention	31	83.8	3	8.1	3	8.1	17	63	4	14.8	6	22.2
Availability of technical assistance	31	83.8	3	8.1	3	8.1	14	51.9	7	25.9	6	22.2
Financial support	21	56.8	11	29.7	5	13.5	6	22.2	10	37	11	40.7
Consulting patient outcomes and progress through the program	31	83.8	4	10.8	2	5.4	20	74.1	3	11.1	4	14.8
Reporting on patient outcomes and progress	33	89.2	2	5.4	2	5.4	21	77.8	1	3.7	5	18.5
Alerts/alarms about patient follow-up in the program	33	89.2	2	5.4	2	5.4	18	66.7	4	14.8	5	18.5

3
